# Prediction and Visualization of Total Volatile Basic Nitrogen in Yellow Croaker (*Larimichthys polyactis*) Using Shortwave Infrared Hyperspectral Imaging

**DOI:** 10.3390/foods13203228

**Published:** 2024-10-11

**Authors:** Sang Seop Kim, Dae-Yong Yun, Gyuseok Lee, Seul-Ki Park, Jeong-Ho Lim, Jeong-Hee Choi, Kee-Jai Park, Jeong-Seok Cho

**Affiliations:** 1 Food Safety and Distribution Research Group, Korea Food Research Institute, Wanju 55365, Republic of Korea; sangseop.k@kfri.re.kr (S.S.K.);; 2 Smart Food Manufacturing Project Group, Korea Food Research Institute, Wanju 55365, Republic of Korea

**Keywords:** hyperspectral imaging, freshness evaluation, total volatile basic nitrogen, spectral pre-processing, chemical imaging

## Abstract

In the present investigation, we have devised a hyperspectral imaging (HSI) apparatus to assess the chemical characteristics and freshness of the yellow croaker (*Larimichthys polyactis*) throughout its storage period. This system operates within the shortwave infrared spectrum, specifically ranging from 900 to 1700 nm. A variety of spectral pre-processing techniques, including standard normal variate (SNV), multiple scatter correction, and Savitzky–Golay (SG) derivatives, were employed to augment the predictive accuracy of total volatile basic nitrogen (TVB-N)—which serves as a critical freshness parameter. Among the assessed methodologies, SG-1 pre-processing demonstrated superior predictive accuracy (R_p_^2^ = 0.8166). Furthermore, this investigation visualized freshness indicators as concentration images to elucidate the spatial distribution of TVB-N across the samples. These results indicate that HSI, in conjunction with chemometric analysis, constitutes an efficacious instrument for the surveillance of quality and safety in yellow croakers during its storage phase. Moreover, this methodology guarantees the freshness and safety of seafood products within the aquatic food sector.

## 1. Introduction

The yellow croaker (*Larimichthys polyactis*) is a protein- and vitamin-rich fish with significant economic, commercial, and nutritional value, and is widely distributed in the East Asian seas [1,2]. However, owing to its high moisture content, neutral pH, and susceptibility to microbial action, oxidation, and enzymatic autolysis, the quality and freshness of the yellow croaker rapidly decline during postmortem storage [3]. Owing to its rapid spoilage, ensuring the freshness and safety of fish is one of the most critical challenges in the aquatic food industry [4,5]. Therefore, it is crucial to determine the freshness of fish to ensure that their consumption is safe and provides appropriate nutrition [6]. Rapid and nondestructive detection methods are essential for evaluating and monitoring the quality and freshness of this valuable and perishable seafood [7,8].

Volatile compounds, including trimethylamine, ammonia, and dimethylamine, which are collectively referred to as total volatile basic nitrogen (TVB-N), are generated through microbial activity and serve as significant indicators for the assessment of the quality and safety of seafood products [9]. Conventional quality assessment methodologies, such as steam distillation, chromatography, and flow-injection analysis, yield reasonably accurate quantitative data regarding the TVB-N concentration, which is an essential parameter for determining the freshness of fish. Nevertheless, these methodologies are characterized by their time-intensive nature and the necessity for skilled personnel, rendering them less advantageous for commercial utilization [10]. Consequently, there exists a pressing requirement to formulate and implement rapid, cost-effective, and user-friendly techniques for the evaluation of fish freshness.

Conventional techniques, such as physicochemical analysis, sensory evaluation, rheological assessments, and electrical measurements, have been employed to assess fish and other seafood [11]. Notwithstanding their notable precision, these techniques generally necessitate costly and intricate instrumentation alongside labor-intensive sample preparation protocols. This constraint hampers their applicability in swift and high-throughput evaluations of aquatic products. Optical sensing methodologies, encompassing spectroscopy and imaging, have been devised to appraise the quality of whole fish and fish fillets. These methodologies present straightforward, expeditious, economical, and non-destructive alternatives to traditional approaches. Among these, hyperspectral imaging (HSI) has emerged as a formidable instrument for the inspection of food and agricultural products [12] and has been utilized in the quality assessment of fish and other seafood [13]. Reflectance measurement constitutes the sole HSI modality employed in the extant literature and is predominantly executed within the visible near-infrared (400–1000 nm) and near-infrared (900–1700 nm) wavelength ranges. HSI, characterized by its non-destructive and rapid nature, excels in the evaluation of food quality and safety [14,15,16]. Furthermore, this technique has been utilized to analyze various indicators of fish freshness, including trimethylamine, total volatile basic nitrogen (TVB-N) [17,18], thiobarbituric acid reactive substances [19], total viable count [20,21,22], and sensory attributes [23,24]. Throughout storage, fluctuations in the TVB-N concentration of yellow croakers induce several chemical transformations that can be elucidated through HSI data. Although the efficacy of HSI in evaluating TVB-N concentrations in fish has been substantiated, enhancing its predictive capabilities remains a compelling area of investigation.

In this investigation, we establish a HSI system aimed at assessing the chemical characteristics of the yellow croaker and improving the precision of its quality forecasting. The principal aims included the creation of an HSI system to analyze the chemical properties of the yellow croaker throughout the storage period; the comparison of the predictive efficacy of various linear methodologies, such as partial least squares regression (PLSR) and multiple linear regression, for the evaluation of total volatile basic nitrogen (TVB-N); and the visualization of freshness indicators represented as concentration images.

## 2. Materials and Methods

### 2.1. Sample Preparation

The yellow croaker (*Larimichthys polyactis*) used in the experiment were all similar in size, with an average length of 20 to 25 cm and a width of around 10 cm. it was acquired in October 2022 from a seafood market located in Busan, Republic of Korea, and was transported in Styrofoam containers accompanied by ice. Upon their arrival, the yellow croakers were preserved at a temperature of 0 °C. The yellow croakers were categorized into six distinct groups, determined by their duration of storage; for chemical and hyperspectral imaging (HSI) analyses: (1) stored for 0 h (utilized immediately), (2) stored for 12 h, (3) stored for 24 h, (4) stored for 36 h, (5) stored for 48 h, and (6) stored for 60 h. Each group comprised 20 yellow croakers, and a total of 120 yellow croakers were utilized to collect hyperspectral data employing an HSI system operating within the shortwave infrared (SWIR) spectrum of 900–1700 nm.

### 2.2. TVB-N Measurement

The total volatile bases nitrogen (TVB-N) concentrations of 120 yellow croaker specimens (20 per designated storage duration) were quantified through an adaptation of the Conway microdiffusion technique [25]. When comparing with TVB-N experimental data, muscle samples were taken from the equatorial region of each yellow croaker, homogenized, and used to ensure consistency and comparability. An approximate mass of 10 g from each specimen was accurately measured and transferred into two distinct beakers. Fifty milliliters of distilled water were incorporated into each beaker, and the resultant mixture was thoroughly agitated and permitted to rest for a duration of 30 min prior to the filtration process. The resultant filtrate underwent neutralization with a 5% sulfuric acid solution and was subsequently adjusted to a specific volume utilizing distilled water, thereby preparing it as a testing solution. The diffusion apparatus was positioned at a slight incline, and precisely 1.00 mL of the testing solution was meticulously introduced into the outer chamber. Following this, 1.00 mL of 0.01 N sulfuric acid was dispensed into the inner chamber adhering to the same procedural standards. A minimal quantity of a sealing agent was uniformly applied to the interface where the cover was affixed. Approximately 1 mL of a saturated potassium carbonate solution was expeditiously introduced to the upper section of the inner chamber, and the cover was promptly secured with a clip. The diffusion apparatus was gently rotated in multiple orientations while being tilted back and forth in order to facilitate the mixing of the testing solution and the saturated potassium carbonate solution within the inner chamber. The apparatus was subsequently allowed to rest at a controlled temperature of 25 °C for a duration of 1 h. Following this period, the cover was removed, and 10 μL of Brunswik’s reagent was incorporated into the sulfuric acid solution situated in the inner chamber. Two average values were derived through titration with a 0.01 N sodium hydroxide solution using a micropipette (a mL). A control experiment was also executed wherein distilled water replaced the testing solution, resulting in two supplementary average values (b mL). These computations were executed in accordance with Equation (1).

(1) $$\mathrm{TVB-N}\;(\mathrm{mg}/100\;g)=0.14\;\times \frac{\left(b-a)\times (f\right)}{W}\times 100\times d,$$

where *W* is the sample weight (g), f is the titer (0.01 N-NaOH, a is the volume of the test solution (mL), b is the volume of the blank solution (mL), and d is a dilution factor.

### 2.3. Hyperspectral Image Acquisition

An HSI system (IMEC Kapeldreef 75, 3001 Heverlee, Belgium) functioning within the shortwave infrared (SWIR) spectrum of 900–1700 nm and exhibiting a resolution of 2048 × 1088 pixels was employed to acquire hyperspectral imaging data. The imaging apparatus was utilized in a line-scan configuration (push broom), whereby a step motor was actuated following the exposure duration of the camera to quantify the reflectance intensity of the acquired image at intervals of 5 nm through a 30 μm aperture. During the hyperspectral image acquisition, the distance between the lens and the moving platform was set to 135 mm, and the platform’s horizontal movement speed was 270 mm/s. In total, approximately 246 distinct spectral bands were recorded. The acquisition rate of frames during spectral collection was set at 10 s, coinciding with an exposure duration of 10 s. Each specimen was positioned on the lateral side and subjected to measurement across 20 iterations. To minimize the effects of varying illumination and detector sensitivity, we used a spectrometer equipped with an indium gallium sensor and three halogen lamps (6 units, 150 W) strategically installed on either side of a dome-shaped illumination source (dimensions: 550 mm × 240 mm × 220 mm). The indium gallium sensor offers high sensitivity and low noise, making it suitable for high-speed imaging. Additionally, before measuring each sample, we performed a calibration using a white reference plate to account for any variations caused by lighting or environmental factors, ensuring the reliability of the hyperspectral imaging results. To mitigate background interference, the spectral data corresponding to each object’s image were delineated utilizing the principal component analysis (PCA)-based region of interest (ROI) functionality available in perClass Mira software (version 3.0.7, perClass BV., Delft, The Netherlands).

### 2.4. Data Analysis

#### 2.4.1. Chemometrics

Multivariate statistical analysis encompasses unsupervised learning, which identifies patterns or interrelations within data when the attributes remain unspecified, and supervised learning, which forecasts outcomes by determining the optimal model through an algorithm configured with both input and output parameters [26]. In this investigation, a Partial Least Squares Regression (PLSR) model was employed to predict the Total Volatile Base Nitrogen (TVB-N) content in yellow croakers. PLSR is a robust supervised learning technique that establishes a linear relationship between the predictor variables, X (spectral data), and the response variable, Y (measured TVB-N content) [27]. Through the application of various spectral pre-processing methods and implementation of the PLSR model, the accuracy of the TVB-N prediction was enhanced. The analysis was conducted utilizing Unscrambler statistical software (version 10.5; CAMO, Trondheim, Norway) [28].

The predicted value of Y was calculated using the following equation:

Y = βX + b,(2)

where β is the vector of regression coefficient and b is the model offset.

Utilizing 20 measurements per group (120 hyperspectral data points pertaining to the freshness of the yellow croaker), the complete dataset of samples was partitioned in a 7/3 ratio, with 90 samples allocated to the development of the model and 30 samples designated for the validation of the model. The classification accuracy of the discrimination model is quantitatively articulated as the percentage (%) of correct classifications within the entirety of the validation dataset. Thirty specimens of yellow croaker (five from each respective storage duration) were employed for the prediction of TVB-N. To assess the efficacy of all constructed PLS models, the coefficient of determination (R_c_^2^) was computed for the calibration model; alongside the root mean square error of calibration (RMSEC), the coefficient of determination (R_cv_^2^) for the cross-validation model, and the root mean square error of cross-validation (RMSECV) were determined.

#### 2.4.2. Spectrum Pre-Processing

To eliminate spectral noise and minimize errors introduced by the sample characteristics, various pre-processing techniques, including standard normal variate (SNV), multiple scatter correction (MSC), and the Savitzky–Golay derivative (SG), were applied. SNV and MSC specifically addressed the removal of light scattering within the spectrum [29]. Moreover, SG was employed to correct baseline deviations due to changes in optical path length or measurement conditions, thereby enhancing the spectral features of trace components [30]. The accuracy of five different groups, comprising both raw and pre-processed data, was evaluated.

#### 2.4.3. Statistical Analysis

TVB-N and acid value data were subjected to one-way analysis of variance (ANOVA), followed by Duncan’s multiple range test for significance evaluation, utilizing SPSS Statistics 20.0 software (IBM Deutschland GmbH, Ehningen, Germany).

#### 2.4.4. Visualization for Chemical Imaging

To visualize the TVB-N content in yellow croakers, the monitoring system was developed using Evince software (ver. 2.7.21, Prediktera AB, Umea, Sweden). Additionally, a PLS model was created within the Evince software using a calibration dataset, and representative predictive images for the distribution map based on the TVB-N content were analyzed.

## 3. Results and Discussion

### 3.1. Spectral Features of Mean Spectra of the Yellow Croaker

**Figure 1:**
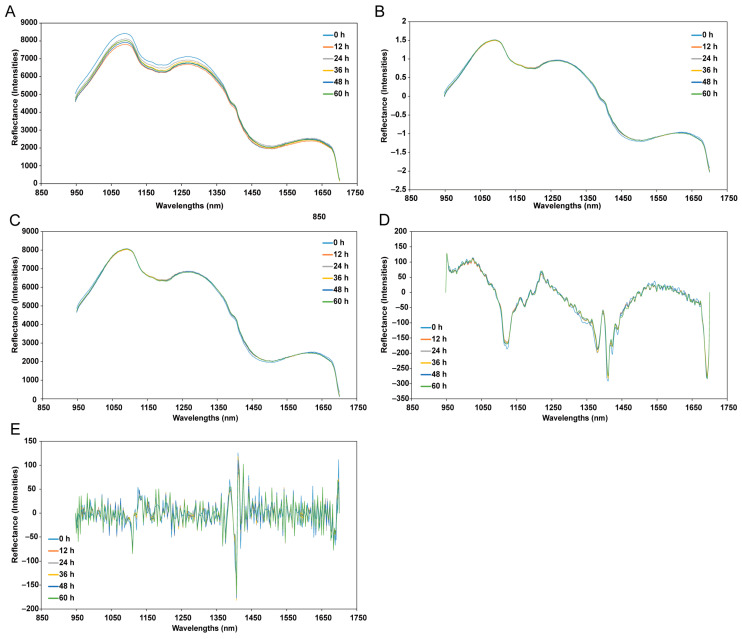
Mean spectra of yellow croakers stored for different intervals in the shortwave infrared (SWIR) band region (900–1700 nm). (**A**), raw spectra; (**B**), SNV; (**C**), MSC; (**D**), SG-1; (**E**), SG2.

Figure 1 illustrates the average spectra (900–1700 nm) of yellow croakers under different spectral pre-processing methods throughout the storage period. Spectroscopy relies on the absorption of light by molecules [31], making it a valuable tool for gaining insights into the chemical composition and molecular bonds within fish tissue. The goal of spectral pre-processing is to eliminate instrument-related noise and reduce the variability in spectra caused by scattering and other factors [32]. In this study, we employed several widely used spectral pre-processing techniques: SNV, MSC, SG1, and SG2. SNV and MSC are scatter correction methods that help minimize the variability between samples due to light scattering [33]. The SG method is effective in reducing random noise; the first derivative corrects baseline drift, while the second derivative effectively eliminates both baseline drift and linear trends [33]. Typically, the first or second derivative is combined with SG. The choice of a specific pre-processing technique remains ambiguous when a single method shows good performance, and it is also unclear which methods should be combined and how they should be applied when a single method is insufficient. Consequently, users often resort to trial and error experiments to explore different options and identify the most suitable method for their dataset. In this study, we adopted this traditional approach to examine these individual pre-processing methods and their combinations. The spectral reflectance curves of samples from different storage days showed similar patterns. However, as time progressed, there was an overall increase in spectral reflectance across the entire investigated wavelength range. The variation in the amplitude of spectral reflectance was evident in the spectral plots of the samples, which is primarily attributed to the chemical and physical changes in fish muscle during freshness degradation [34,35] driven by microbial and enzymatic activities. Consequently, fresher samples with lower TVB-N levels exhibited lower reflectance curves, and vice versa.

### 3.2. PLS Score Plots for Classifying the Freshness of Yellow Croaker According to Its Storage Periods

**Figure 2:**
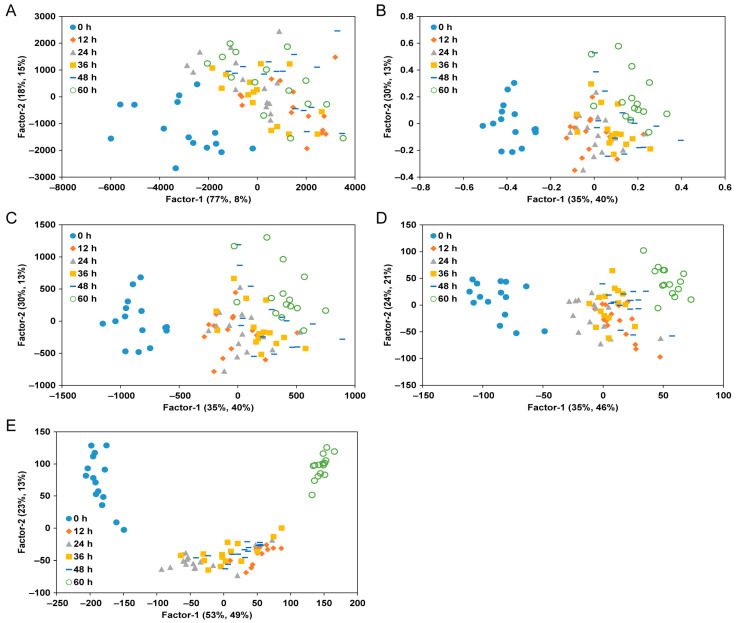
PLS score plots for classifying the freshness of yellow croaker according to storage periods. (**A**), raw spectra; (**B**), SNV; (**C**), MSC; (**D**), SG-1; (**E**), SG-2.

The original reflectance spectral data matrix was transformed into a coordinate system where the samples were positioned according to their PCA scores rather than their intensity in wavelength space [36]. As a result, samples with similar spectral characteristics tended to cluster in the same location within the principal component space. The score plots depicted in Figure 2, which represent the TVB-N content and spectral pre-processing methods in two dimensions based on principal component factors derived from hyperspectral spectra, demonstrate a clear differentiation. In these plots, samples stored for 0, 12, 24, 36, 48, and 60 h are represented by blue circles, red rhombuses, gray triangles, yellow squares, blue bars, and green circles, respectively. For raw, SNV, and MSC data, it was challenging to categorize samples according to their storage period, except during the initial stage (0 h). However, in the case of SG-1 and SG-2, indicated by the dotted circle, the samples were distinctly classified according to the spectral pre-processing method applied to assess yellow croaker freshness. SG-1 displayed a distinct separation and grouping from other pre-processing methods after 60 h of storage. SG-1 exhibited clearer grouping than other pre-processed samples stored for 60 h, with SG-2 showing even greater separation than SG-1. This observation aligns with previous findings indicating that SG is the most effective spectral pre-processing method for fish, including tuna [37]. Consequently, score plot analysis using hyperspectral data from the SWIR region can effectively illustrate differences in the distribution of TVB-N characteristics in yellow croakers. This plot, however, only demonstrates qualitative differences among the samples without considering their quantitative attributes [36].

### 3.3. Changes in TVB-N of Yellow Croaker According to Freshness

The TVB-N measurements used as indicators of freshness are presented in Table 1. TVB-N content is a key metric for evaluating fish freshness. Initially, the yellow croaker exhibited a TVB-N content of 4.41 ± 2.04 mg/100 g, signifying freshness. However, this value increased significantly over time, reaching 24.76 ± 7.17 mg/100 g. Typically, TVB-N levels in fresh fish, moderately spoiled fish, fish in the early stages of spoilage, and completely spoiled fish are 5–10 mg/100 g, 15–25 mg/100 g, 30–40 mg/100 g, and above 50 mg/100 g, respectively [38]. After 48 h of storage, the mean TVB-N content in the yellow croaker was 17.85 mg/100 g, indicating moderate spoilage. Based on this critical threshold, the acceptable shelf life of the yellow croaker was determined to be 48 h. This distinction between fresh and stale samples during storage was crucial in developing a reliable calibration model for predicting the TVB-N content, which in turn allows for the estimation of the shelf life and quality of the yellow croaker. According to Duncan’s multiple range test, with a significance level set at 0.05, the TVB-N values varied significantly depending on storage duration.

### 3.4. Comparison of Prediction Models Using Different Spectral Pre-Processing Methods

Table 2 presents the results of model validation using the PLSR model, where different pre-processing methods were employed to enhance the accuracy of the quality analysis. To construct a robust model, the conventional approach involves combining spectral pre-processing with a target model, such as partial least squares (PLS), for classification and prediction [39]. Therefore, we identified the most accurate method by comparing raw data with data processed through SNV, MSC, SG-1, and SG-2. The R_c_^2^ for SNV and MSC pre-processing methods were 0.8544 and 0.8534, respectively, indicating that both methods contributed similarly to the model’s predictive accuracy for TVB-N levels, suggesting they are equally effective in reducing the effects of light scattering. The SG first and second derivative pre-processing methods were applied to prevent spectral baseline shifts caused by the optical path or analytical conditions, or to emphasize micro characteristics. Their corresponding R_c_^2^ values were 0.9028 and 0.8603, respectively. Among the pre-processing methods, SG-1 yielded the highest accuracy. The R_cV_^2^ values for all pre-processing techniques ranged from 0.7912 to 0.7991; however, SG-1 demonstrated the highest value at 0.8265, while SG-2 showed the lowest at 0.7699.

### 3.5. Prediction Results for TVB-N Contents of Yellow Croakers According to the Spectral Pre-Processing Methods

**Figure 3:**
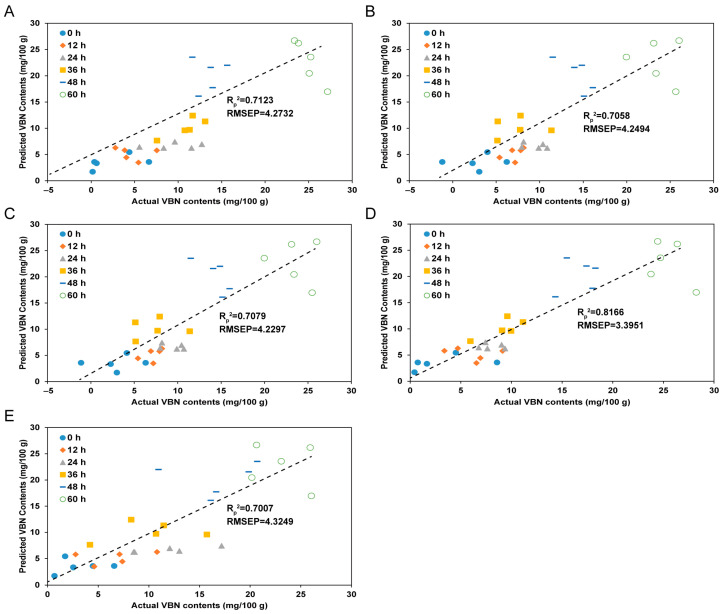
Accuracy of predicting TVB-N in yellow croaker by partial least-squares models using various pre-processed hyperspectral spectra. (**A**) raw, (**B**) SNV, (**C**) MSC, (**D**) SG-1, and (**E**) SG-2.

Figure 3 illustrates the predicted TVB-N content in yellow croakers using PLS regression models with various spectral pre-processing methods. The prediction accuracy (R_p_^2^) of the TVB-N content models was 0.7123 (without pre-processing), 0.7058 (SNV), 0.7079 (MSC), 0.8166 (SG-1), and 0.7007 (SG-2), showing a trend consistent with the results in Table 2. Notably, the accuracy of the TVB-N prediction model for yellow croakers was significantly improved by the SG-1 pre-processing method. This finding aligns with previous research [40,41]. The SG method, integral to SG-1, effectively reduces noise and smoothens spectral data, resulting in cleaner data that are crucial for enhancing predictive accuracy. Prior studies have demonstrated that SG smoothing markedly improves classification accuracy while reducing baseline drift and tilt in hyperspectral data [42]. Moreover, SG-1 pre-processing enriches spectral information by preserving critical spectral features while minimizing irrelevant noise. This enhancement aids in distinguishing subtle differences in spectral features, which is vital for the accurate classification and prediction in hyperspectral imaging applications [40]. The increased accuracy of TVB-N prediction using SG-1 in this study can be attributed to its effectiveness in correcting signal noise caused by the surface reflectance of the yellow croaker, while also preserving the distinct spectral patterns associated with freshness. This makes SG-1 the most suitable pre-processing method. Similar observations have been reported in other studies involving samples with significant light reflectance [43]. The superior performance of SG-1 can be attributed to its capacity to mitigate the effects of light scattering and baseline drift, which are common challenges in hyperspectral imaging, particularly in samples such as fish that exhibit high surface reflectance. By enhancing the quality of spectral data, SG-1 facilitates more accurate predictions of freshness indicators such as TVB-N, which are crucial for assessing the quality and shelf-life of fish during storage. These findings suggest that integrating SG-1 pre-processing into hyperspectral imaging systems for real-time monitoring of fish freshness could significantly enhance the reliability and efficiency of quality control processes in the seafood industry. Future research could focus on refining this approach by combining other freshness indicators, such as the K-value, with TVB-N to develop a more comprehensive model for evaluating both early and late stages of spoilage.

### 3.6. Chemical Imaging of Freshness Information of Yellow Croakers

**Figure 4:**
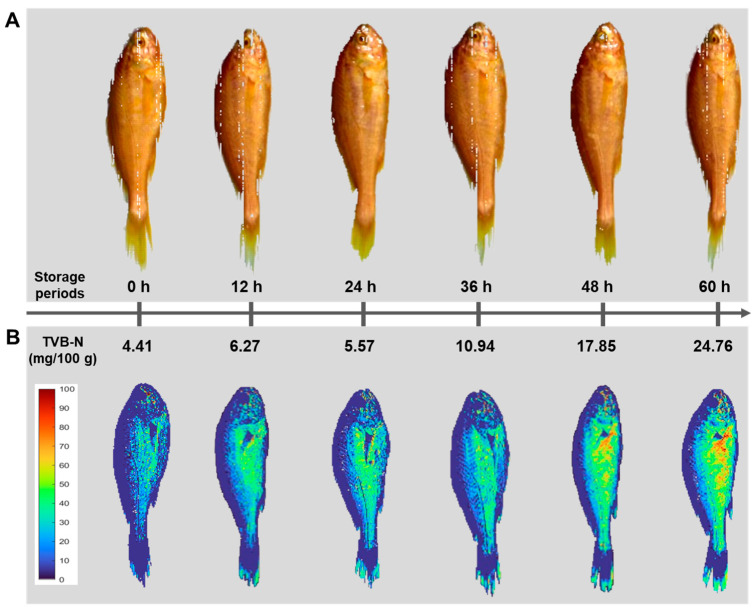
Chemical imaging of freshness according to the storage periods of the yellow croaker. (**A**) original RGB images, (**B**) hyperspectral based chemical imaging for the prediction of TVB-N contents.

Chemical imaging of a freshness indicator (TVB-N) as a pseudocolor map is the final and most important stage in HSI systems. Such a pixel-wise map is the main advantage of HSI over traditional spectroscopy [17]. Figure 4 shows four distribution maps of the yellow croaker at different storage periods (0, 12, 24, 36, 48, and 60 h). In this figure, the changes in the TVB-N values are indicated sample by sample and even location by location within the same sample. A linear color scale shows the quality status of the yellow croaker at various locations during storage. In this color bar, blue represents the best quality (low values of TVB-N) and red represents the worst quality of the yellow croaker. Because of the varying rates of chemical and microbial degradation of various compounds, the freshness and quality at different locations within a sample are not uniform. The distribution of colors in the map was not homogeneous, indicating varying degrees of TVB-N in different parts of the yellow croaker. For example, initially, most pixels were dark blue, indicating the high quality (low TVB-N values) of the yellow croaker. During the spoilage process, the color of the pixels changed, and by the last day of storage, most of the pixels turned red. As shown in Figure 4, the central parts of the fish samples generally displayed a lower quality (yellow-red color). This is because the center of the fish was highly reflective of the height and scale of the sample. The predicted TVB-N values were 0.41 (0 h), 5.37 (12 h), 13.23 (24 h), 14.93 (36 h), 18.11 (48 h), and 31.02 (60 h) mg/100 g, with actual TVB-N values of 3.60, 6.27, 7.45, 14.93, 16.09, and 20.43 mg/100 g, respectively. As shown in Table 1, the TVB-N value increased with storage time. Therefore, this visualization method is reliable and can provide spatial information on the TVB-N value in the yellow croaker. This capability exemplifies one of the advantages of an HSI system, which can effectively visualize and display the distribution of target components. Consequently, the HSI system, especially in the SWIR region, could be used for the prediction of quality according to the storage period of the yellow croaker and could also be used as a monitoring system for the accumulation of TVB-N.

## 4. Conclusions

This study highlighted the effectiveness of using an HSI system in conjunction with various spectral pre-processing methods to assess the freshness of the yellow croaker by predicting its TVB-N content. Among the pre-processing techniques tested, the SG-1 method demonstrated the highest prediction accuracy and significantly enhanced the performance of the PLSRF models. The SG-1 technique was particularly adept at correcting signal noise from surface reflectance while preserving the unique spectral patterns that indicate the freshness of the yellow croaker, making it an ideal choice for applications involving significant light reflectance. These results align with previous studies that have reported similar improvements in model accuracy using SG-1 pre-processing. The use of pseudocolor maps to visualize freshness indicators provided a clear spatial distribution of TVB-N within the samples, further underscoring the potential of HSI as a non-destructive, rapid, and reliable tool for monitoring seafood quality and safety during storage. This approach offers considerable benefits to the aquatic food industry by ensuring the freshness and safety of seafood products. Overall, the integration of HSI with chemometric analysis proves to be a powerful method for the real-time assessment and monitoring of fish quality, with potential applications that could extend beyond yellow croakers to other seafood and perishable food products.

## Figures and Tables

**Table 1:** Changes in the TVB-N content of yellow croaker according to storage periods.

Storage Periods	0 h	12 h	24 h	36 h	48 h	60 h
TVB-N (mg/100 g)	4.41 ± 2.04 ^a,1^	6.27 ± 1.81 ^a^	5.57 ± 2.28 ^a^	10.94 ± 1.31 ^b^	17.85 ± 3.21 ^c^	24.76 ± 7.17 ^d^

^1^ Different letters in the same row are significantly different (*p* < 0.05).

**Table 2:** Calibration and cross-validation values in accordance with spectral pre-processing using the PLSR model.

Spectrum Pre-Processing ^1^	Calibration (*n* = 90) Parameters ^2^			
	R_c_^2^	RMSEC	R_cv_^2^	RMSECV
Raw	0.8395	3.4496	0.7991	3.8525
SNV	0.8544	3.2857	0.7975	3.9497
MSC	0.8534	3.2963	0.7912	4.0116
SG-1	0.9028	2.6842	0.8265	3.6853
SG-2	0.8603	3.2182	0.7699	4.1698

^1^ SNV, standard normal variate; MSC, multiple scatter correction; SG-1, Savitzky–Golay 1st derivative; SG-2, Savitzky–Golay 2nd derivative. ^2^ R_c_^2^, correlation coefficient of calibration; RMSEC, root mean square error of calibration; R_cv_^2^, correlation coefficient of full cross-validation; RMSECV, root mean square error of cross-validation.

## Data Availability

The original contributions presented in the study are included in the article, further inquiries can be directed to the corresponding author.

## References

[1] Ren J.S., Jin X.S., Yang T., Kooijman S.A.L.M., Shan X.J. (2020). A dynamic energy budget model for small yellow croaker: Parameterisation and application in its main geographic distribution waters. Ecol. Model..

[2] Shao Y.Y., Shi Y.K., Wang K.L., Li F.F., Zhou G.Y., Xuan G.T. (2023). Detection of small yellow croaker freshness by hyperspectral imaging. J. Food Compos. Anal..

[3] Hassoun A., Sahar A., Lakhal L., Aït-Kaddour A. (2019). Fluorescence spectroscopy as a rapid and non-destructive method for monitoring quality and authenticity of fish and meat products: Impact of different preservation conditions. LWT Food Sci. Technol..

[4] Chen H.Z., Zhang M., Bhandari B., Yang C.H. (2020). Novel pH-sensitive films containing curcumin and anthocyanins to monitor fish freshness. Food Hydrocoll..

[5] Zhao X., Chen L., Wongmaneepratip W., He Y., Zhao L., Yang H.S. (2021). Effect of vacuum impregnated fish gelatin and grape seed extract on moisture state, microbiota composition, and quality of chilled seabass fillets. Food Chem..

[6] Fazial F.F., Tan L.L. (2021). Phenylalanine-responsive fluorescent biosensor based on graphene oxide-chitosan nanocomposites catalytic film for non-destructive fish freshness grading. Food Control.

[7] Song Y.J., Lyu C.C., Guo W. (2018). On-line monitoring research of seafood quality safety based on image monitoring. J. Coast. Res..

[8] Khoshnoudi-Nia S., Moosavi-Nasab M. (2019). Prediction of various freshness indicators in fish fillets by one multispectral imaging system. Sci. Rep..

[9] Özoğul F., Özoğul Y. (2000). Comparison of methods used for determination of total volatile basic nitrogen (TVB-N) in rainbow trout (*Oncorhynchus mykiss*). Turk. J. Zool..

[10] Sun W., Li H., Wang H., Xiao S., Wang J.H., Feng L. (2015). Sensitivity enhancement of pH indicator and its application in the evaluation of fish freshness. Talanta.

[11] Hassoun A., Karoui R. (2017). Quality evaluation of fish and other seafood by traditional and nondestructive instrumental methods: Advantages and limitations. Crit. Rev. Food Sci. Nutr..

[12] Qin J.W., Kim M.S., Chao K.L., Chan D.E., Delwiche S.R., Cho B.K. (2017). Line-scan hyperspectral imaging techniques for food safety and quality applications. Appl. Sci..

[13] Cheng J.H., Sun D.W. (2014). Hyperspectral imaging as an effective tool for quality analysis and control of fish and other seafoods: Current research and potential applications. Trends Food Sci. Technol..

[14] Cheng J.H., Sun D.W. (2015). Rapid quantification analysis and visualization of loads in grass carp fish flesh by hyperspectral imaging method. Food Bioprocess Technol..

[15] Sun D.W. (2010). Hyperspectral Imaging for Food Quality Analysis and Control.

[16] Ghidini S., Varrà M.O., Zanardi E. (2019). Approaching authenticity issues in fish and seafood products by qualitative spectroscopy and chemometrics. Molecules.

[17] Cheng J.H., Sun D.W., Wei Q.Y. (2017). Enhancing visible and near-infrared hyperspectral imaging prediction of TVB-N level for fish fillet freshness evaluation by filtering optimal variables. Food Anal. Methods.

[18] Cheng J.H., Sun D.W., Zeng X.A., Pu H.B. (2014). Non-destructive and rapid determination of TVB-N content for freshness evaluation of grass carp (*Ctenopharyngodon idella*) by hyperspectral imaging. Innov. Food Sci. Emerg. Technol..

[19] Dai Q., Cheng J.H., Sun D.W., Zeng X.A. (2015). Advances in feature selection methods for hyperspectral image processing in food industry applications: A review. Crit. Rev. Food Sci. Nutr..

[20] Wu D., Sun D.W. (2013). Hyperspectral imaging (TS-HSI) for non-invasive determination of microbial spoilage of salmon flesh Potential of Time Series. Talanta.

[21] Cheng J.H., Sun D.W. (2015). Rapid and non-invasive detection of fish microbial spoilage by visible and near infrared hyperspectral imaging and multivariate analysis. LWT Food Sci. Technol..

[22] Khoshnoudi-Nia S., Moosavi-Nasab M., Nassiri S.M., Azimifar Z. (2018). Determination of total viable count in rainbow-trout fish fillets based on hyperspectral imaging system and different variable selection and extraction of reference data methods. Food Anal. Methods.

[23] Cheng J.H., Sun D.W. (2015). Data fusion and hyperspectral imaging in tandem with least squares-support vector machine for prediction of sensory quality index scores of fish fillet. LWT Food Sci. Technol..

[24] Wang X., Shan J.J., Han S.Q., Zhao J.B., Zhang Y.T. (2019). Optimization of fish quality by evaluation of total volatile basic nitrogen (TVB-N) and texture profile analysis (TPA) by near-infrared (NIR) hyperspectral imaging. Anal. Lett..

[25] Conway E.J., O’Malley E. (1942). Microdiffusion methods. Ammonia and urea using buffered absorbents (revised methods for ranges greater than 10 mug. N) (revised methods for ranges greater than 10 μg. N). Biochem. J..

[26] Lee K., Park S., Sung S., Park D. (2019). A study on the prediction of CNC tool wear using machine learning technique. J. Korea Converg. Soc..

[27] Jang H.J., Choi C.H., Choi T.H., Kim J.H., Kwon G.H., Oh S.I., Kim H., Kim Y.J. (2016). The analysis of oat chemical properties using visible-near infrared spectroscopy. Korean J. Agric. Sci..

[28] Lewis-Beck M.S., Bryman A., Liao T.F. (2004). The SAGE Encyclopedia of Social Science Research Methods. Encyclopedia of Social Science Research Methods.

[29] Wu D., Sun D.W. (2013). Advanced applications of hyperspectral imaging technology for food quality and safety analysis and assessment: A review—Part II: Applications. Innov. Food Sci. Emerg..

[30] Zhang Z., Song X., Chen Y., Wang P., Wei X., Tao F.L. (2015). Dynamic variability of the heading-flowering stages of single rice in China based on field observations and NDVI estimations. Int. J. Biometeorol..

[31] Choudhry P. (2016). High-throughput method for automated colony and cell counting by digital image analysis based on edge detection. PLoS ONE.

[32] Cui C., Fearn T. (2018). Modern practical convolutional neural networks for multivariate regression: Applications to NIR calibration. Chemom. Intell. Lab. Syst..

[33] Rinnan Å., Berg F.v.d., Engelsen S.B. (2009). Review of the most common pre-processing techniques for near-infrared spectra. TrAC Trends Anal. Chem..

[34] Xiong Z., Sun D.W., Pu H., Xie A., Han Z., Luo M. (2015). Non-destructive prediction of thiobarbituricacid reactive substances (TBARS) value for freshness evaluation of chicken meat using hyperspectral imaging. Food Chem..

[35] Xu J.L., Riccioli C., Sun D.W. (2016). Efficient integration of particle analysis in hyperspectral imaging for rapid assessment of oxidative degradation in salmon fillet. J. Food Eng..

[36] Kamruzzaman M., ElMasry G., Sun D.W., Allen P. (2011). Application of NIR hyperspectral imaging for discrimination of lamb muscles. J. Food Eng..

[37] Sánchez-Parra M., Fernández Pierna J.A., Baeten V., Muñoz-Redondo J.M., Ordóñez-Díaz J.L., Moreno-Rojas J.M. (2024). Rapid screening of tuna samples for food safety issues related to histamine content using fourier-transform mid-infrared (FT-MIR) and chemometrics. J. Food Eng..

[38] Song H.N., Lee D.G., Han S.W., Yoon H.K., Hwang I.K. (2005). Quality changes of salted and semi-dried mackerel fillets by UV treatment during refrigerated storage. Korean J. Food Cook Sci..

[39] Mishra P., Biancolillo A., Roger J.M., Marini F., Rutledge D.N. (2020). New data preprocessing trends based on ensemble of multiple preprocessing techniques. TrAC Trends Anal. Chem..

[40] Kim S.S., Choi J.Y., Lim J.H., Cho J.S. (2023). Non-destructive quality prediction of domestic, commercial red pepper powder using hyperspectral imaging. Korean J. Food Preserv..

[41] Wang L., Pang L., Yan L., Zhang J. (2022). Nondestructive rapid identification of soybean varieties using hyperspectral imaging technology. J. Appl. Spectrosc..

[42] Zou Z., Chen J., Wu W., Luo J., Long T., Wu Q., Wang Q., Zhen J., Zhao Y., Wang Y. (2023). Detection of peanut seed vigor based on hyperspectral imaging and chemometrics. Front. Plant Sci..

[43] Xu F., Huang X., Tian X., Yu S., Zhang X., Zareef M. (2024). Application of hyperspectral imaging and colorimetric sensor array coupled with multivariate analysis for quality detection during salted duck eggs processing. J. Food Process Eng..

